# Help-seeking before and after episodes of self-harm: a descriptive study in school pupils in England

**DOI:** 10.1186/1471-2458-8-369

**Published:** 2008-10-24

**Authors:** Sarah Fortune, Julia Sinclair, Keith Hawton

**Affiliations:** 1 University of Oxford Centre for Suicide Research, Department of Psychiatry, Warneford Hospital, Oxford, OX3 7JX, UK

## Abstract

**Background:**

Deliberate self-harm in young people is a cause for concern in many countries. The vast majority of episodes of self-harm do not result in presentation to hospital and relatively little is known about to whom or where adolescents who harm themselves go for help.

**Methods:**

This school-based survey of 5,293 15–16 year olds in the United Kingdom investigated sources of help and barriers to help seeking before and after an episode of self-harm.

**Results:**

Friends (40%) and family (11%) were the main sources of support. Far fewer adolescents had sought help from formal services or health professionals. Barriers to help seeking include perceptions of self-harm as something done on the spur of the moment and therefore not serious or important or to be dwelt upon. Many adolescents felt they should be able to, or could cope on their own and feared that seeking help would create more problems for them and hurt people they cared about or lead to them being labelled as an 'attention seeker'. The decision to seek help was in some cases hampered by not knowing whom to ask for help. Gender and exposure to self-harm in the peer group influenced perceived barriers to help-seeking.

**Conclusion:**

There are both push and pull factors' acting on young people in their understanding of what leads them to want to harm themselves and potential mechanisms for seeking help. The implications for community based prevention programmes are discussed.

## Background

There have been several studies of deliberate self-harm (DSH) in adolescents based on those who have presented to hospital [1-3], which have largely focused on methods of DSH, psychiatric disorders and problems faced by adolescents. There have also been two recent large-scale community-based surveys of self-harm in adolescents in England [4,5]. These have shown that the prevalence of self-harm is much more frequent than is indicated by hospital presentation. In a study from the Centre for Suicide Research in Oxford, in which more than 6,000 15–16-year-olds in a representative sample of 41 schools were surveyed, 7% (11% girls and 3% of boys) reported an act of self-harm in the previous year that met the study criteria [5]. In only 13% of these cases had self-harm resulted in hospital presentation. The finding that the majority of episodes of self-harm do not lead to hospital-based care has been replicated in other countries [6,7].

Relatively little is known about to whom or where adolescents who engage in self-harm or have thoughts of self-harm go for help. In the Oxford schools study noted above the pupils reported that friends were their main source of support. This was true of those who had self-harmed or thought about doing so, as well as other pupils. However the reliance on friends relative to other sources of support was relatively greater among those who had thought about, or actually harmed themselves compared with other pupils [8]. The use of friends as the main source of support is developmentally appropriate. However, among young people who self-harm this creates a paradox because a strong direct association exists between individual and peer self-harm [9]. Having a friend who has self-harmed in the previous year is associated with increased rates of thoughts of self-harm and actual self-harm among both girls and boys [10].

There are mixed findings as to whether or not seeking help from friends and family increases the opportunities for getting help from more formal sources such as health professionals. In a longitudinal study of young adults in New Zealand who had self-harmed the use of informal support structures such as family and friends was associated with significantly higher rates of using formal support structures, such as health services [11]. On the other hand, some young people seek help from peers, but do not go on to access more formal help, particularly if they have poor skills in managing their emotions [12], or if their peers are also suicidal [13].

Many young people who harm themselves do not access any form of support, or are unwilling to seek help from certain sources, and it is important to understand the reasons for this. Barriers to accessing help identified in previous studies include cognitive, attitudinal and practical factors that appear to vary by gender and ethnic groups. Problematic cognitions and problem solving strategies were identified by Gould et al., [13], who reported that among American high school pupils those at highest risk of suicide were more likely to endorse help-avoidant strategies such as believing you should be able to sort your problems out on your own, and less likely to endorse help-seeking strategies, such as getting advice from a friend. Similarly, Nada-Raja et al., [11] found that among adolescents who had harmed themselves some felt it was not necessary to consult anyone because of a belief that problems will work themselves out, or because of a belief that no one can really help.

Help negation has been defined as the refusal to accept or access available help as a function or manifestation of patient hopelessness, pessimism or cynicism regarding treatment [14]. This characteristic suggests that those most in need of help may find it most difficult to access it. In a community study of 14 – 18 year olds, higher levels of suicide ideation were associated with lower levels of intention to seek help [15]. In a further study hopelessness, gender and prior help-seeking experience did not have any greater influence than suicide ideation in reducing intentions to seek help [16], leading the authors to conclude that suicide ideation decreased intention to seek help and acts as a barrier to help seeking, especially from mental health professionals and telephone help lines. They also concluded that it may contribute to ineffective problem-solving.

Several studies have identified attitudinal barriers to seeking help in self-harm [17]. Young people are concerned about being considered 'weird' or 'crazy' if they seek help from mental health services [18]. There are also gender differences in help-seeking. In a recent study in England distressed young men aged 16 – 24 years were less likely to seek help of any kind including from their friends or family and were significantly more distressed than their female counterparts when they did eventually seek help from their GP [19]. Similarly, two thirds of male 15 – 19 year-old suicide attempters presenting to hospital in Finland had no healthcare contacts in the month before the acts, and half had no healthcare contacts subsequently [20].

Previous experiences of help-seeking may have an impact on future decisions to seek help [21]. Between 20 and 30 thousand adolescents per year attend hospital in the UK following an episode of DSH [10]. Some studies have indicated that many adolescents tend to perceive contact with hospital services as unhelpful due to poor continuity of care and their perception that hospital staff have difficulty relating to young people [22] together with negative expectations about therapy [23]. In contrast, Burgess et. al, [3] found that adolescents who had self-harmed who were offered therapy following discharge from hospital, and agreed to be followed up, rated it positively.

Models of help seeking for health-related difficulties involving several stages have been proposed. These build on Butcher and Crosbies' [24] work which outlined four stages: the perception of the problem, motivation to act, the perception that something can be done and finally, the decision to act. Later models proposed by Greenley and Mullen [25] and Murray [21] elaborated on the process by which people define their experiences as problematic and legitimate, and consider potential sources of help.

A small number of previous studies have documented the most frequently used sources of help by adolescents who have engaged in DSH. However, very few have examined the perception of self-harm as a problem among these young people, whether they believe help seeking will be beneficial, and the impact that this has on their motivation to seek help. An exploration of these factors is the aim of this study.

The school-based survey from the Centre for Suicide Research mentioned above included questions about help seeking and barriers to help seeking. The responses to these questions form the basis of this study which extend previous publications on the prevalence of suicidal phenomena, reasons for self-harm and adolescents' views on how to prevent DSH [5,8,26,27]. The research questions addressed by the current study are:

• What sources of help do adolescents with a lifetime history of self-harm approach before and after an episode of DSH?

• What are the barriers to seeking help before and after an episode of DSH?

• How do adolescents perceive self-harm and how may this influence help-seeking behaviour?

• Do findings regarding help seeking differ according to gender, exposure to DSH by peers, or previous personal experiences of help-seeking as a result of DSH?

Based on the answers to these questions, we present a conceptual model of help-seeking behaviour in young people following DSH

## Methods

The study was conducted in a representative sample of 41 secondary schools in Oxfordshire, Northamptonshire and Birmingham. Full details of the method have been provided elsewhere [5,10]. The main aim of the study was to determine the prevalence of DSH in England and factors associated with it. Pupils, mainly aged 15 – 16 years, filled in a self-report, anonymous questionnaire that took approximately 20 – 30 minutes to complete. The questionnaire included demographic information (gender, age and ethnicity) and questions about lifestyle, life events, problems, thoughts of self-harm, DSH, and coping, plus scales to measure depression, anxiety, impulsivity, and self-esteem. DSH was defined as an act with a non-fatal outcome in which an individual deliberately did one or more of the following, initiated behaviour (for example, self cutting, jumping from a height), which they intended to cause self harm; ingested a substance in excess of the prescribed or generally recognised therapeutic dose; ingested a recreational or illicit drug that was an act that the person regarded as self harm; ingested a non-ingestible substance or object.

In addition, all respondents who had acknowledged a history of DSH were asked to mark from a list of nine potential sources of help all those that they had contacted either before and/or after the most recent episode of DSH. Those who had engaged in DSH but had not sought help were asked about barriers to help seeking: "Why didn't you try to get help *before *you took the overdose or tried to harm yourself" and "Why didn't you try to get any help *afterwards *for the problems that led you to take an overdose or try to harm yourself".

The study was explained to students by researchers or teaching staff two weeks prior to the study and again by researchers on the survey day. Parents were informed by letter about the study and were asked to inform the research team if they did not want their child to participate. Students were given the choice to participate. The study was conducted in accordance with the guidelines of the British Educational Research Association and approved by the Oxfordshire Psychiatric Research Ethics Committee.

### Data analysis

Qualitative research methods were used to identify themes occurring in response to the open-ended questions. Individual pupil responses were classified to allow identification of the key themes and quantification of their extent.

Initially an open coding scheme was used, whereby responses were analysed and a preliminary code assigned either to an actual word or phrase from the text (e.g. I was scared) or a more general topic (e.g. embarrassed/shame). These codes were then further refined and a clear description characterising each one generated. Codes were then applied to all other related passages of text. This method, which utilises some of the principles of grounded theory [28], was repeated until a comprehensive coding scheme was well matched to the free text. Computer software [29] aided the systematic analysis of the data, facilitating the comparison of emerging themes with already coded responses, thereby preventing the development of ideas not borne out by the data. The coding scheme was developed by the research team in an iterative fashion. Using the coding manual generated by the team, JS then recoded 24% of responses, blind to the original coding decisions. This process yielded good inter-rater reliability, with agreement in 87% – 93% of items.

The qualitative responses of the adolescents were analysed first for the overall sample and subsequently by the following variables: gender, ethnicity, those with or without friends who had engaged in DSH or died by suicide, and those who had ever attended hospital following DSH. Examples of relevant quotations will be used to illustrate some of the themes identified. Some quotations have been modified in order to ensure anonymity of respondents.

## Results

**Figure 1:**
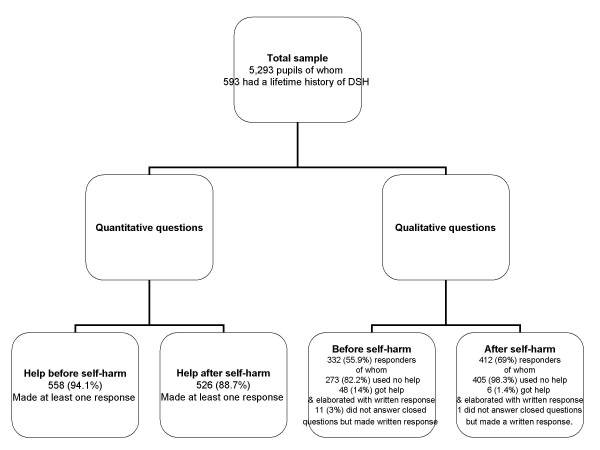
**Flow chart of respondents**.

The sample of 41 schools which participated in the study included 35 comprehensive schools, 4 independent and 2 grammar schools, of which nine were single sex schools. Of the 6020 pupils who took part in the study 5293 (87.9%) completed all the questions on DSH, of these 10.3% had a lifetime history of DSH. The breakdown of the pupils' responses regarding the quantitative and open-ended questions is outlined in Figure 1. Adolescents who responded to the open-ended questions were not significantly different, in terms of gender, ethnicity or exposure to DSH, from those who did not respond. Nearly three quarters of respondents were female (n = 242), 88% were white and 59% had been exposed to DSH among their peers. The methods of DSH among respondents were cutting (58%) and overdose (23%), other multiple methods (8%), other single methods (7%), and both cutting and overdose (5%).

### Sources of help before and after episodes of self-harm

The main sources of help contacted by the adolescents before and after their most recent episode of DSH were identified from a list that was provided (see Table 1). There were no significant differences in the sources of help approached between males and females, or between those with and without friends who had engaged in DSH. The most common source of help was friends, followed by family members. Adolescents were nearly four times as likely to seek help from friends following an episode of DSH than family members. Telephone help-lines and teachers were mentioned by smaller numbers of the adolescents.

**Table 1:** Sources of help contacted before and after most recent episode of DSH by adolescents with a lifetime history of DSH

	**Source of help beforehand**			**Source of help afterwards**		
	N = 558*	N = 137*	N = 421*	N = 526*	N = 133*	N = 393*
	N (%)	Male (%)	Female (%)	N (%)	Male (%)	Female (%)
Friend	221 (40)	46 (34)	175 (42)	246 (47)	57 (43)	191 (49)
Someone in your family	58 (11)	10 (8)	48 (12)	118 (23)	25 (20)	96 (25)
Telephone helpline	39 (8)	5 (4)	34 (9)	15 (3)	4 (3)	11 (3)
Teacher	33 (6)	8 (6)	25 (6)	26 (5)	9 (7)	17 (5)
Psychologist/psychiatrist	26 (5)	8 (6)	18 (5)	39 (8)	13 (10)	28 (8)
GP/Doctor	18 (4)	2 (2)	16 (4)	37 (8)	9 (7)	29 (8)
Social worker	16 (3)	7 (6)	9 (2)	20 (4)	6 (5)	15 (4)
Drop-in/advice centre	10 (2)	2 (2)	8 (2)	7 (1)	2 (2)	5 (1)
Any other source	45 (9)	9 (7)	36 (9)	20 (4)	7 (6)	14 (4)

* N varies due to missing data, N represents the maximum number of responses

Formal sources of help, such as psychologists or psychiatrists or general practitioners were mentioned by many fewer respondents. Adolescents with friends who had engaged in self-harm were more likely to seek help from their friends (65% vs. 35%).

Following DSH the pattern of sources of help were similar except that more adolescents said their source of help was friends (47% vs. 40%), family members (23% vs. 11%), a psychiatrist or psychologist (8% vs. 5%) and a GP or doctor (8% vs. 4%). In part this may reflect formal care offered to those adolescents whose DSH episode came to clinical attention.

### A model of help-seeking behaviour among adolescents who have engaged in DSH

**Figure 2:**
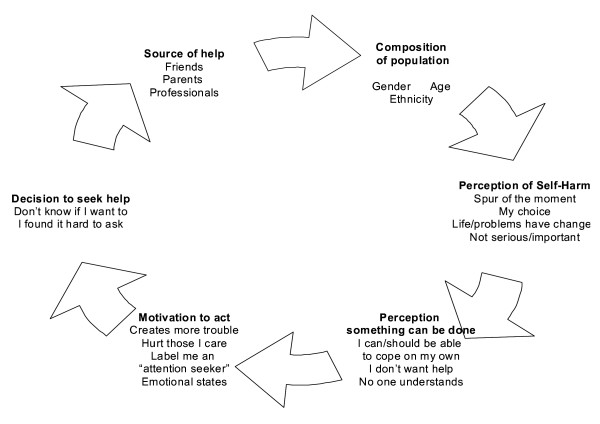
A model of help-seeking behaviour before and after self-harm among adolescents.

Building on previous models of help-seeking behaviour, particularly the work of Murray [21], on the basis of the responses of the adolescents with a lifetime history of DSH we suggest a model of help-seeking which combines factors that operate both before and after an episode of DSH (Figure 2). At the first level the help-seeking behaviours of adolescents who have harmed themselves may be influenced by demographic factors such as their gender, age and ethnicity ('composition of the population' in Figure 2).

In the following section we describe five stages of help seeking and explore sub-themes which occur within each stage based upon responses of the adolescents in this study. The stages are: 1) perceptions of DSH; 2) perception that something can be done; 3) motivations to seek help; 4) barriers to help-seeking and; 5) choosing sources of help.

### Perceptions of the episode of DSH

DSH was described by the participants in this study as having several important dimensions. The perceptions of self-harm as a difficulty or problem for which they could generate an explanation covered four main themes:

#### a. Spur of the moment

Some adolescents (5%, n = 18) described their DSH as a spur of the moment act, a stupid thing or something impulsive, which made it unlikely that they would have sought help beforehand:

"I didn't think about it before I did it. It just happened"

"Because I didn't intend to do it, it wasn't planned and also cause I was alone".

#### b. Not that serious or important

Five percent (n = 17) of adolescents referred to the lack of perceived seriousness of their intent or injury as a reason for not seeking help before the act and 31 (8%) made reference to this theme after the episode:

"I did not feel it was serious enough for help"

"Because it never harmed me that bad and I was sick after"

"It wasn't serious cuts, some of them were just small marks"

Boys were more than twice as likely to comment on this aspect of DSH compared with girls (14%, n = 13 vs. 6%, n = 18).

#### C. My choice

Twenty-six adolescents (8%) said they had been reluctant to seek help prior to their most recent act of DSH because they perceived DSH as something they chose to do and were concerned that others would try to prevent them harming themselves in the future, although this dropped to 4% (n = 15) afterwards:

"If I tried to get help then they would of tried to stop me but that was not what I wanted at the time ..."

"It was a decision I wanted to make, I couldn't cope and still can't, they would of tried to stop it. I wouldn't live with all the problems on my back"

"I wanted to do it. It was social and brought me, my friends and my girlfriend closer together"

"Because I felt I wanted to do something new which I could keep to myself which I felt good about doing".

"Because for the past few years I've been trying to harm myself and if I talked to people about it they will try and get me help – to try and stop me from doing it"

This was a theme mentioned twice as often by boys than girls (12%, n = 11 vs. 6%, n = 15).

#### D. Life or my problems have changed

For some adolescents the episode of DSH had resulted in a change in circumstances or a change in how they felt and therefore they did not seek help afterwards. Others described themselves as having moved on from where things were when they harmed themselves (13%, n = 53):

"Because I realised it was petty and got on with my life"

"The problem resolved itself and I returned to a happy state of mind"

"'Cos the overdose didn't work I was fine the next day I was glad for that. This was ages ago I like my life now"

"I felt I could control myself not that the rage had gone and I felt new and refreshed so no longer needed help".

Girls were more than twice as likely as boys indicate that things had changed following their DSH (15%, n = 46 vs. 7%, n = 7).

Thirty-eight respondents (9%) reported that after they harmed themselves they though it was something they would not do again, they wanted to put it to the back of their minds and felt certain they would not engage in DSH again. This theme was mentioned by similar proportions of boys and girls:

"I had learnt from what I did, never to do that again"

"Because I just wanted to forget it and move on, it wasn't the first time it had happened so I know I could get over it"

"Because it doesn't happen on a regular occasion. One-off"

"Cause it was one off and wouldn't happen ever again cause I ain't no nutta".

### Perception that something can be done

#### A. I can, or should be able to cope on my own or no one can help me

The most prominent responses by adolescents who had not sought help prior to their most recent act of DSH related to coping. Just over one in five adolescents (21%, n = 70) reported that they had not sought help before the act because they felt that they could cope with their feelings and DSH alone, they planned to sort things out themselves, or didn't think they needed help from anybody beforehand. A similar proportion gave such reasons to explain why they had not sought help after the act (23%, n = 93):

"I could get through on my own and I did better than if anyone had helped me"

"Because I was trying to cope with it myself"

Seven percent (n = 23) of adolescents reported they felt no one could help them or that they felt they were beyond help prior to the episode and 3% (n = 11) mentioned this afterwards.

"I don't think anyone can sufficiently help me"

"Because no one can help me only I can help myself that's if I want to"

"I felt beyond help".

Boys were more likely to express this view than girls (10%, n = 9 vs. 6%, n = 15).

#### b. No-one would understand

A small number of adolescents (5%, n = 15) did not seek help before harming themselves or afterwards (3%, n = 11) because they felt people would not listen to them or care about their problems. Girls were more likely to raise this issue than boys (6%, n = 15 vs. 1%, n = 1):

"Because no-one around me cared and that's the reason I did it, sometimes now I still feel the same because my parents never realise I'm there and my friends don't give a shit about me they only care about themselves"

"I only tried to get help but no one listened and it felt like no one cared so I thought if no one cares and I don't care then I might as well just do it".

Seven percent (n = 22) of pupils said they did not seek help prior to an act of DSH because of concerns that people would not take them seriously or would fail to understand why they were self-harming or the experience of harming oneself. This theme was less prominent following the episode (3%, n = 11).

"I thought they would tell me my problems were insignificant and they wouldn't understand how and why I felt like I did"

"I felt I was the only one who knew what I was going through"

"Because I felt as though no one would understand me and that they would think I was being a stupid attention seeker or it was just my hormones etc. I felt no one could see exactly why I was doing it".

Boys and girls mentioned this theme with similar frequency. Pupils with peers who had self-harmed were twice as likely to be concerned that people would not take them seriously or would not understand their DSH behaviour (8%, n = 16 vs. 4%, n = 6).

#### C. Didn't want help

A related, but different issue was the statement by some adolescents that they didn't want help before their act of DSH (17%, n = 58). These responses differed from those in the previous theme because they reflected the idea that the adolescents were aware they might need help, but they did not want it. Adolescents who had taken overdoses (20%, n = 15) or cut themselves (19%, n = 36) were more likely to state they did not want help than those who used other methods of self-harm. Girls were more likely than boys to state that they did not want help (19%, n = 47 vs. 12%, n = 11).

A small proportion of respondents were clear in their own minds that they did not seek help beforehand because they wanted to die (5%, n = 18). Somewhat surprisingly, all of the respondents in this category were girls. This theme was mentioned by only 6 respondents after the act of self-harm:

"Because I didn't want help at that time I just wanted to kill myself"

"Because I didn't want help, I hated myself and I wanted to die".

Eleven respondents (3%) reported that they could not be bothered asking for help beforehand or they didn't see the point or they didn't care anymore.

### Motivation to act

The motivation to act is influenced by the individuals' estimation of the cost benefit ratio of taking action and the impact of the emotional states on the motivation to act.

#### A. I will hurt or worry people who I care about

Some adolescents were put off seeking help beforehand because of fears they might have worried or hurt others (3%, n = 11) a similar proportion gave this as the reason for not seeking help afterwards (4%, n = 16). Girls were more concerned about this than boys (4%, n = 10 vs. 1%, n = 1):

"I don't really like talking to people I don't know or trust about my problems. My mum had enough problems of her own and I didn't think my friends would understand how serious I felt about my life"

"I didn't want to bother anybody with my problems. I'm happy go lucky person who had just bit a rough patch".

#### B. It could create more trouble

Some adolescents (4%, n = 12) had negative expectations about helping organisations and what help might involve, or they felt that seeking help would make their situation worse (3%, n = 11 before, 1%, n = 5 afterwards):

"This was because I felt that I would make the situation worse. And anyway I really did want to die"

"Cause it'd mess things up even more"

"I didn't need the hassle and I didn't want my parents to know about it"

#### C. Emotional states

Adolescents said emotions such as feeling scared, stupid or alone meant they were not able to seek help prior to harming themselves. Seventeen adolescents (5%) described feeling scared and afraid to ask for help:

"I was scared to ask"

"I was scared so I only spoke to a friend, he told me not to do it and that he was scared".

Twelve adolescents (4%), all girls, said they felt they were being stupid or that people would think they were stupid if they asked for help before:

"Because they would just think I was stupid and tell my mum who would screw at me"

"....parents would think I was being stupid".

A small number of respondents (3%, n = 10) described how alone they felt prior to the act, in addition to actually being alone when they harmed themselves:

"I felt alone and I didn't want any help I just wanted to escape"

"I couldn't think straight and I was alone in the house and too upset to talk to anyone"

"Because I was really angry and I wanted to be alone".

#### D. Being labelled as an 'attention-seeker'

Eleven adolescents (3%), of whom ten were girls, did not seek help before harming because they feared being labelled or dismissed as an 'attention seeker' or that people would attribute attention seeking as the only reason they had engaged in DSH:

"Felt the same response would always be met 'she did it for attention"'

"I was frightened people would think I was just trying to get sympathy and attention which I wasn't"

"Because people might think I was a stupid little girl who just wanted attention. I didn't think anyone could help me then".

Feeling ashamed or embarrassed following an episode of DSH, or being concerned other people would label them as crazy, was mentioned by 6% (n = 25) respondents, of whom 24 were girls:

"I was embarrassed and scared and ashamed"

"Because I didn't want people to say I was a psycho".

A related concern among some adolescents (4%, n = 14) was the desire for their feelings and DSH to be a secret and that no one should know about it stopped them seeking help before and 6% (n = 24) afterwards. This theme was more frequently mentioned by girls than boys (5%, n = 12 vs. 2%, n = 2).

"I didn't want anyone to know"

"Because my friend only found out when she saw the cuts (by accident) I still tried to hide it after that".

### Decision to seek help

The adolescent accounts indicated that even once the decision to seek help had been made, some barriers still persisted.

#### A. Found it hard to talk

A small group of adolescents (6%, n = 19) described their difficulty in finding the words to communicate their distress, not knowing what to say and not wanting to talk about their problems beforehand and 2% (n = 10) afterwards:

"I'm not good at talking to people. I like to keep my thoughts and feelings to myself. A problem shared is still a problem ...."

"....it was difficult to explain to anyone why I was feeling the way I was"

"Didn't know how to tell someone I needed help".

#### B. Didn't know what to do

Ten respondents (3%) commented that they didn't know what to do, who to approach or where to get help from prior to the episode of self-harm:

"I didn't know who or how to get help"

"Because I didn't know if or who could help".

### Choice of source of help

The following section includes a summary of factors relevant to the sources of help chosen by adolescents based on their responses to the open ended questions.

#### A. Friends

Friends were mentioned as a source of help after an episode (6%, n = 24), and more frequently by girls than boys (7%, n = 21 vs. 3%, n = 3). The responses included reference to friends helping the adolescent, friends recognising their distress or the adolescent telling their friends about their DSH behaviour:

"My friends finally recognised my emotions and helped, listened"

"... my boyfriend also helped me as he has done the same before".

Adolescents who had been exposed to DSH among peers were more likely to comment about friends compared with those not exposed to DSH among peers (8%, n = 20 vs. 3%, n = 4) and their comments were more likely to include specific references to the DSH of their friends:

"I had learnt from what I did, never to do that again. My boyfriend also helped me as he has done the same before"

"Because it was all my own opinions. I thought that everyone would think I was being stupid or looking for attention. It was too hard to tell anyone. I did tell one friend who had tried to kill himself".

#### B. Parents

Twenty adolescents (5%), all girls, mentioned their parents after the most recent episode of self-harm. They commented on fears that their parents would find out about their DSH or the true extent of their suicidal ideas, worries that they would hurt their family through their behaviour or in some cases being afraid of their parents. Those without DSH in their peer group were more likely to comment about their parents (7%, n = 11 vs. 4%, n = 9).

"Because my mother did not want to acknowledge that I was in a depressed state of mind so encouraged me never to mention the episode and my situation rapidly improved so I felt I did not need help"

"I saw the doctor and said I was over it and told my family it was a cry for help not a serious attempt. If I told the truth it would've hurt them too much for me to handle"

"Because after that I realised how stupid it was, how upset my family would be"

"Because me and my mum are very close now and I can tell her everything. We worked through it together".

#### C. Professionals

Health professionals were mentioned by a very small number of respondents (2%, n = 7), some being adolescents who were in treatment with mental health professionals but did not want them to find out about their DSH behaviour:

"It was none of anyone else's business. I was having counselling for my anorexia but I did not tell the psychiatrist or social worker or counsellor"

"I thought that they would tell my psychologist".

Following an episode of self harm only a small number of pupils commented on health professionals, although this was more frequent among girls (3%, n = 9 vs. 1%, n = 1). Some comments reflected that the DSH had led to help from a professional. Others commented that they were afraid of professionals, or had considered talking to a health professional, but had not done so:

"My mother found out. She thought I needed help – took me to the GP who referred me to a psychologist who helped me"

"I didn't really know what the problem was and still is. I lie in bed at night and get this sort of craving to cut myself. I don't know why. Plus I am terrified of medicine, needles, doctors, hospital"

"I wanted to tell my doctor but then I was scared that he'll tell my parents".

A small number of adolescents (3%, n = 12) made reference to the hope that their DSH would be noticed by someone and that it would, or did, result in someone reaching out to help them:

"I didn't think anyone could help me. I didn't want to go to them, I wanted them to see I was upset and come to me"

"I got help after I cut myself not after the overdose"

"Because my friend helped me and stopped me before I took too many".

### Previous help-seeking experiences

Of the 104 respondents who had attended hospital following an episode of DSH, 46 made some form of qualitative response about help beforehand and 50 made a response regarding reasons they had not sought help following an episode of DSH. These responses were assessed in terms of comments relating to previous help-seeking behaviour.

An evaluation of the responses among those who had not sought help before their last episode of DSH yielded very few comments that can be attributed to previous experiences of help. Only three participants made comments about previous experiences of trying to get help:

"Could not be arsed with a doctor saying nothing is the matter"

"I thought that they would tell my psychologist"

"I only tried to get help but no one listened and it felt like no one cared so I thought if no one cares and I don't care then I might as well just do it".

## Discussion

In this study we have investigated the sources of help adolescents approached before and after an episode of DSH and the barriers to seeking help based on information collected in a large community based sample of adolescents. In line with previous studies [6,11,12], friends and family were described by adolescents who had engaged in DSH as being the main sources of support both prior to, and following an episode of DSH. Far fewer adolescents had sought help from formal services or health professionals. The quantitative data in this study are important in understanding the absolute numbers of adolescents who seek help from particular sources but do not tell us very much about the processes or experiences of adolescents who harm themselves and make choices about seeking assistance. The qualitative aspect of this study provides complementary insight into the process of help seeking.

Adolescents with a lifetime history of DSH, the majority of whom had not sought help before or after their most recent episode of DSH, were asked why this was. A model of help-seeking in relation to self-harm behaviour is proposed which extends previous studies [e.g. [21,24,25]] by examining the application of these models in community rather than clinical settings [25] and with a larger and more representative sample of participants [21]. Our study extends previous work by focusing on help-seeking relating to self-harm rather than for more generic problems and reporting of actual experiences, which may be more accurate than intentions.

This study is one of the few that has investigated a sample of adolescents in this way in the community. However, several limitations of the study should be borne in mind. First, pupils who did not attend school on the day of the survey were not included. There are also a considerable number of those present who did not complete the entire questionnaire. These selection biases reduce the generalisability of these findings as do the small sample sizes in some sub-analyses. The dependence on pupils writing down their responses limited our ability to explore nuances of the qualitative responses compared with an interview study. The experiences that participants related are subject to recall biases including variations in the time elapsed between the episode of self-harm they were recalling and the date of the study.

An important aspect of this study is enhancing our understanding of how adolescents in the community perceive self-harm beyond studies that have been conducted with frequent repeaters and/or clinical samples [30,31]. The perception among respondents of self-harm as a difficulty or a problem for which there is an explanation differed from the views often held by adults and health professionals. The adolescents' often described DSH as something that they did on the spur of the moment and by implication it was therefore not serious or important. For some, DSH was something they chose to do and that they were concerned that others would try and prevent them from doing. However, some responses demonstrated the sequelae, both practical and psychological, of the most recent episode of DSH. Nearly one in ten adolescents reported that once they had harmed themselves they felt this was not something they would do again and they wanted to put it to the back of their minds. A smaller number, particularly male respondents, felt that their intent or injury from the DSH was not that serious so they didn't think it was important to get help. Of concern is research that suggests that adolescents are not always good at judging the potential lethality of methods of DSH [31,32], and while they may report low suicidal intent they can select relatively lethal methods and vice versa. The effect of self-harm described by adolescents in this study was their sense that things had changed following the act. Some adolescents, particularly girls, described a change in circumstances, others felt differently after the DSH and yet others described a general sense of things having moved on. It is possible that these adolescents are at risk of future DSH as things are not resolved and they have not added to their repertoire of coping behaviours. Yet their immediate crisis is over and they may not be motivated to seek help. On the other hand this narrative may reflect a process of growing out of DSH [31].

In terms of help-seeking following DSH, the next stage of our proposed model is the perception as to whether something can be done [21,24]. The most prominent theme expressed by adolescents was the belief that they could cope on their own, that they planned to sort things out themselves and didn't feel they needed help from anyone. This theme was mentioned by a similar number of adolescents as a reason for not seeking help either before or after their most recent episode of DSH. Boys were more likely to perceive that they could cope on their own than girls. This narrative represents a challenge for effective interventions and appears to act as a barrier to help-seeking behaviour both before and after episodes of DSH. It is already known that help-seeking is particularly problematic for males [e.g. [20,33]] and boys feeling they can cope on their own may add to our understanding of factors which have an impact on their help-seeking behaviour. It is also interesting that adolescents have this perspective when others may argue that the fact they are engaging in DSH behaviour suggests they are not coping well. Previous studies suggest that DSH is associated with poor coping and poor problem solving in adolescents [34]. In addition some adolescents in this study expressed concerns that people would not take them seriously or would fail to understand why they were self-harming, as well more negative cognition that no one could help them or they were beyond help.

Another prominent theme for why adolescents did not seek help before they engaged in DSH was the idea that they did not want help, even if on one level they might have been aware that they needed help (or were ambivalent about asking for help). Girls were more likely to state that they did not want help. A surprising finding was that all the respondents who stated they did not want help because they wanted to die were girls (although the numbers are small). This is contrary to previous research that has suggested that boys generally have greater intent to die than girls [2] and have higher rates of suicide [35]. It is possible that the act of DSH somehow shifted the adolescents' belief that they did not need help, as it was mentioned by many fewer pupils as something that had prevented them seeking help afterwards. In combination, these two themes of individuals believing that they do not need help, and that they do not want help, are powerful cognitive factors that warrant further investigation.

There is evidence from this study that the motivation to act [21,24] and to seek help before or after an episode, has several elements. Girls were particularly anxious about being labelled as an 'attention seeker' or dismissed as 'stupid' by others if they asked for help. It is possible that these responses reflect the perception that cutting, particularly by girls, is a feminine behaviour which reflects failure [2,33]. It is also possible that the recent focus in the media on young people who cut themselves and the interpretation of this behaviour as of relatively low lethality and low intent to die, has inadvertently alienated some adolescents who are harming themselves using other methods or who do not consider their own behaviour as having this particular meaning in their own lives. Concerns about being labelled as 'crazy' were more prominent among those who did not have peers who self-harmed. This may reflect the acceptability of some behaviours in certain peer groups and the normal adolescent's desire to not stand out from their peer group.

Some adolescents suggested they were deterred from seeking help by the concern that it would actually make things worse or that they would hurt people they loved. Feeling alone, stupid and scared reflected the complex feelings that some adolescents had about their DSH behaviour and the difficulties they face when weighing up the costs and benefits of asking for help. Boys were more likely to express these views. In addition, even among those who wanted help, a number of adolescents said they didn't know who to try and get help from, in addition to finding it difficult to talk about their problems.

## Conclusion

In line with previous research, there appear to be both push and pull factors acting on young people in their understanding of what leads them to want to harm themselves and potential mechanisms for seeking help. In contrast to existing literature [22], prior experiences of help did not emerge strongly from this data as having an influence on helpseeking behaviour. This may reflect the way in which adolescents recall and relate their experiences or it may be that the study method was not well suited to exploring this issue. Similarly, relatively few differences were apparent between adolescents from different ethnic groups and this requires further investigation. Barriers to help seeking are often considered in practical terms, such as lack of transport or finding it hard to make an appointment. However, in this study most of the perceived barriers to help seeking were attitudinal and were located either within the individual (e.g. I can cope on my own) or in relation to others, such as worrying what other people will think of them.

This study has important implications for the prevention of self-harm by adolescents. First, given that the majority of episodes of self-harm in this age-group do not result in presentation to medical or mental health services, the delivery of effective community-based prevention programmes is extremely important in addition to efforts to improve clinical services for people who harm themselves. School-based programmes promoting psychological well-being are one method of attempting to reach adolescents who harm themselves [e.g. [36]]. The findings of this study suggest these prevention programmes need to address the gap between perceptions of self-harm held by adolescents and service providers on dimensions including the seriousness and importance of these acts. Secondly, the promising interventions for preventing depression in school students [37] could be incorporated into self-harm interventions to address the finding that a number of young people have depressive cognitions such as "no one can help me" and "I should be able to cope on my own" and poor problem solving skills. The fact that adolescents who self-harm turn to their friends for support is to be expected however the ways in which having peers who self-harm enhances or hinders further help-seeking behaviour requires further study. The role of family factors in self-harm is well established and effective prevention efforts need to balance the importance of respecting autonomy and confidentiality for young people and while also managing adolescents' concerns that getting help for their self-harm may make things worse for them or their family.

This study indicates that we need further research on barriers to help seeking, both among people who have never sought help for their DSH, and among those who have received services.

## Competing interests

The authors declare that they have no competing interests.

## Authors' contributions

KH conceived of the study, participated in its design and coordination and preparation of the manuscript. SF and JS conducted the statistical analysis and interpretation of the data. SF was the lead in drafting and revising the manuscript, JS contributed to this process. All authors have read and approved the final manuscript.

## Pre-publication history

The pre-publication history for this paper can be accessed here:


